# Exploring patient safety risk in an emergency ward for substance use through a mixed-method analysis

**DOI:** 10.1186/s12913-024-10621-z

**Published:** 2024-01-31

**Authors:** Jakob Svensson

**Affiliations:** Division of Risk Management and Societal Safety, Lund University, Box 118, Lund, SE-22100 Sweden

**Keywords:** Patient safety, Emergent risk, Healthcare management, Psychiatry, Case study

## Abstract

**Background:**

There is limited knowledge of how psychiatric patient safety measures can detect and understand risk as an emergent property within a healthcare system. Overcrowding poses a risk for patient safety in psychiatric emergency wards and is associated with increased mortality and violence. This paper aims to explore patterns of risk emergence in a psychiatric ward and provide insights into the dynamics of workload pressure.

**Method:**

A case study was conducted in a psychiatric emergency ward for patients with substance use disorders. The study employed a four-phased mixed-methods design. Phase one used clinical experts to identify patient safety pressure issues. Phase two used data on patient visits extracted from medical records between 2010 and 2020. In phase three, a quantitative analysis of patient visits and diagnosis was made. Phase four used a focus group of clinical experts for a semi-structured interview, analysing the result from phase three.

**Result:**

Trend analysis demonstrated a steady growth of patient visits to the emergency ward over the studied ten-year period. The findings showed a decrease in patients being diagnosed with delirium when visiting the emergency ward and an increase in percentage of patients receiving a psychosis diagnosis. The focus group expressed concerns about delayed treatments, increased violence and underestimating patients’ needs.

**Conclusion:**

This study indicated that increased workload pressure can be predicted at a system level by analysing patient visits and diagnostics trends over time. The study advocates for ongoing awareness of patient safety risks by monitoring factors identified by clinical front-end workers as potential sources of risk. Healthcare management could employ supportive tools to detect and address emerging risks, including expected workload, overcrowding, staffing issues or bed shortages.

## Background

There is limited knowledge of how safety measures in psychiatric care can detect and understand risk as an emergent property within a healthcare system [1, 2]. The emergence of risk refers to the process by which unforeseen or previously unrecognized risks become apparent at a system level, often evolving over time due to interactions at lower system levels [3–5]. In this paper, Rasmussen’s (1997) theory of natural migration of activities towards increasing risk of accidents [6] has inspired the study of how front-end workers respond to workload pressure. The theory refers to the normal changes over time occurring in the daily work as a result of competing priorities. This means the process by which potential threats or uncertainties shift from one area to another.

Efforts to strengthen patient safety are generally made through quality improvements such as best practices and standardisation [7], and as these measures in psychiatry seldom focus on single target shortages [1], the complexity of patient safety, which entails dimensions of changing working conditions and pressure over time, is overlooked [8].

Overcrowding in the emergency department can be defined as an imbalance of the need for emergency care and the hospital’s availability to provide that service [9]. It is a problem concerning the entire healthcare system in a clinic [10], and overcrowding limits correct, timely and efficient hospital care [9]. Safety theory of complex systems, such as psychiatric healthcare, suggests that patient safety include an ability to monitor, adapt and respond to changing situations [4, 8, 11]. Overcrowding introduces such situations and poses a risk for patient safety in psychiatric emergency wards [12], being associated with increased mortality [13, 14] and violence [15, 16].

The traditional view of patient safety can be defined as an absence of accidents and incidents [17]. However, formalised practices sometimes struggle to eliminate adverse events [18]. Patient safety could encompass the capability to foresee healthcare needs [19]. This strategic approach integrates everyday work to support healthcare performance variability, allowing healthcare professionals to adapt their actions based on risk [20]. Performance variability, in this context, refers to the ability to adapt under various conditions, and is suggested as a prerequisite in understanding why things go right in healthcare [21].

An emergency ward can be affected by performance pressure where daily routines is controlled by management to cope with increasing demand for healthcare [22].Flexibility can support adaptive capacities that enables front-end workers to cope with uncertainty. The ability to adjust can create safety in an uncertain environment [21], and front-end workers can use their adaptive capacities to respond to emerging risks [17]. Emergent risk implies levels of aggregation and time scales, and can be argued to be a system property, such as overcrowding, which effect patient safety on greater scale [3, 5].

An emergency ward can be used as an example to study emergent risk within the healthcare system [23]. This means considering risk as a result of normal interactions on a micro level, which has consequences for the system level [3–5]. This paper aims to explore patterns of risk emergence in a psychiatric ward and provide insights into the dynamics of workload pressure.

## Method

### Study setting and design

A case study was conducted in a psychiatric emergency ward for patients with substance use disorders. In a case study, both qualitative and quantitative data can be collected to help build a comprehensive understanding of a case [24, 25], and case studies are well suited when studying complexity and system changes over time in healthcare [25, 26]. Thus, this study addresses patient safety via the case of a psychiatric healthcare unit. The study used a mixed-methods design, inspired by a sequential explanatory design [27]. This approach integrates both quantitative and qualitative methods in a specific sequence, which is particularly useful when a researcher aims to explore complex phenomena in healthcare [28]. The design encompasses a quantitative phase in which the researcher collects and analyses numerical data. The insights obtained from the quantitative phase subsequently guide the qualitative phase, in which the researcher collects and analyses non-numerical data such as interviews, observations or documents.

The planning phase involved defining precise research objectives, selecting methods through literature review, and conducting expert probing for iterative refinement. The research design was planned with a supervisor, which included continuous engagement in peer consultations to ensure valuable insights were incorporated in the qualitative process. The quantitative data collection, including statistical analyses, was discussed with a statistician at the studied clinic to uphold result validity.

The purpose of the quantitative compilation of variables in this study was to visualize trends in what the front-end workers had identified as risk in patient safety. The quantitative phase aimed to identify trends and patterns in patient visits, contributing to the understanding of changes in patient volume over a ten-year period.

### The case

The studied emergency ward for substance use disorders is open 24/7 and offers and initiates treatment for people over the age of 18 with addiction to alcohol, narcotics, other drugs and/or pharmaceuticals in Stockholm County, Sweden. The ward has 9 ordinary and 3 extra beds and approximately 20,000 visits per year, of which half of the visitors come via the police or ambulance. The ward offers both voluntary care and care in accordance with the forced healthcare laws (Act on Treatment of Addicted Persons, Act on Compulsory Psychiatric Care). The ward is the only county-wide emergency department that provides healthcare according to forced healthcare laws for patients with addiction and dependency disorders, and it provides healthcare assessment, treats risky withdrawal conditions and monitors recovery for patients with substance use disorders.

On arrival, patients are assessed (triaged) by a nurse or nurse-assistant before being enrolled. The triage function means that the nurse makes an immediate medical assessment of the patient’s condition on arrival. This initial assessment guides the initial prioritisation, followed by a medical examination and diagnostics and a decision on further treatment. Immediately after the first assessment, the patient can be admitted for a few hours or up to a few days to become sober, followed by a few days of withdrawal treatment if the criteria for care are met (e.g., risk of complicated withdrawal). Some patients need medical assessments before being admitted to a treatment home, social services accommodation or requested consultation from another clinic.

### Data collection

The study consisted of a four-phase design.

#### Phase one

Clinical experts from the studied clinic were probed to validate which parameters should be included for the statistical analysis. They were asked what they considered a patient safety challenge and to give an example of a challenging day. Key informant sampling was used to recruit the participants, and they were selected based on their knowledge and expertise of the studied ward [29]. The qualitative responses from the experts were transformed into quantitative variables by using transcripts of key words.

The first phase also included an overview of relevant documents for the ward to provide further insight into the healthcare system that could be relevant to the interviews in phase four [30]. Documents that related to the ward’s mission and purpose together with their embedded units were selected. The documents included internal reports and evaluations, planning documents and policies.

#### Phase two

Data collection instruments was designed, along with sources of data and data selection points, to ensure data accuracy and reliability. Data on patient visits to the studied ward were extracted from medical records between 2010 and 2020. Based on answers from the clinical experts in phase one, dates and times of patient visits to the studied ward, patients’ psychiatric diagnoses and patients’ genders were collected. No exclusion was made, and no other information was gathered for quantitative analysis. Data collected in this phase formed the basis for the interpretation in Phase three and four.

#### Phase three—data analysis

A quantitative analysis of patient visits was made by coding in R, which is a free software environment for statistical analysis. The coding structure were iteratively developed as data (gender, time, day and diagnosis) were added. In phase one, psychosis and delirium tremens were identified as the two diagnoses with the greatest impact on staff resources, and these diagnoses were singled out for further evaluation. To differentiate between types of diagnoses, the psychiatric taxonomy of ICD-10 was used, and the analysis included diagnoses of psychosis (F10.5, F11.5, F12.5, F13.5, F14.5, F15.5, F16.5, F17.5, F18.5, F19.5 and F29.9) and delirium tremens (F10.4). This selective inclusion allowed for a more targeted analysis of a specific manifestation within the broader spectrum of substance-related disorders. The statistical analysis included trend analyses and moving averages.

The integration of quantitative and qualitative data was achieved through a convergent design [31], where quantitative results was used to inform the selection of participants for follow-up qualitative interviews in phase four.

#### Phase four

With the content of the statistical analysis, a semi-structured interview was conducted using a focus group of clinical experts. To ensure the reliability and relevance of information, the sampling strategy to select clinical experts was guided by their work experience where all had worked 10 years or more in the clinic. The sampling process aimed to capture diverse perspectives, including individuals with varying experience levels and roles. Focus groups can be used to explore a specific topic and involve bringing together a group of experts chosen to meet specific criteria [32]. A pragmatic approach was taken to perform the interview [33] as the everyday work in the psychiatric emergency ward accommodates complexity and is occasionally interrupted. Preliminary outcomes from phase three were discussed within the focus group, and notes from the group discussion were transcribed. The qualitative data were analysed using a thematic coding approach. This involved initial open coding, thematic categorization, and the constant comparison method to identify emerging themes [34]. Triangulation of data occurred by comparing findings from the quantitative and qualitative phases, which offered a deeper understanding of emerging patient safety risks. Thematic analysis helped identify recurring themes and patterns in the responses provided by the clinical experts. This qualitative phase delved into the complexities and challenges faced by the front-end workers and how patient safety was influenced by factors like overcrowding and workload pressure. The utilization of a focus group of clinical experts contributed to a cross-validation of the findings, and an exploration of the patient safety challenges identified in earlier phases, offering insights into the complexity of healthcare system dynamics.

## Results

The broader objective of study was to explore the dynamics of risk emergence in a psychiatric emergency unit, offering insights to patient safety challenges and potential areas for monitoring risk. Table 1 summarizes how many patients visited the emergency ward each year and illustrated a steady increase in patient visits each year.

**Table 1:** Number of visits distributed per year

Year	Number of visits
2010	13,268
2011	13,695
2012	14,042
2013	14,147
2014	14,428
2015	15,446
2016	15,832
2017	16,180
2018	18,099
2019	19,167
2020	19,486

### Patient visits over time

**Fig. 1:**
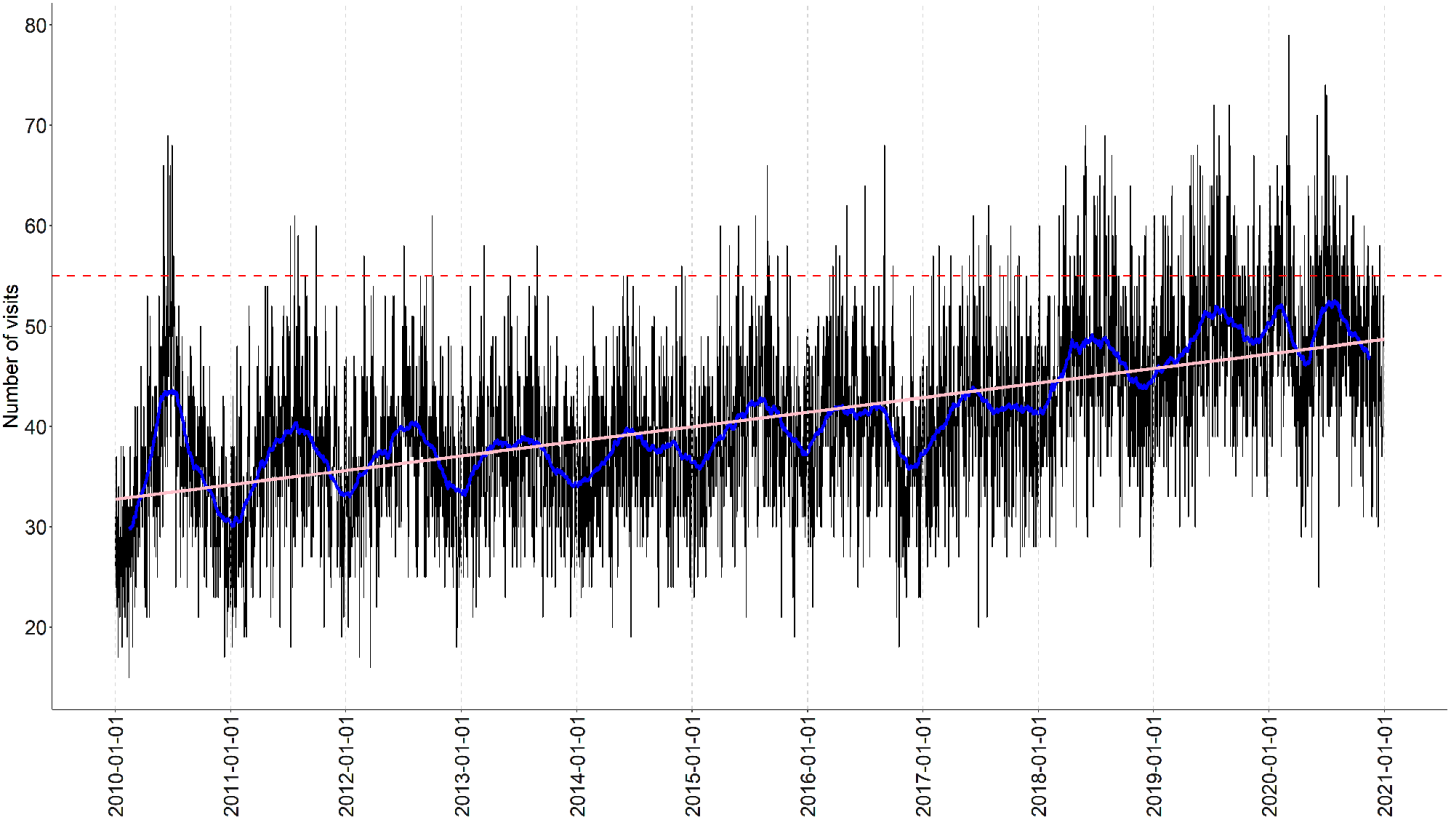
Number of patient visits each day over the studied period. Red dotted line shows the estimated max capacity. Blue line is the moving average, and pink line is the trend

Fluctuations in patient visits occurred daily, but a trend analysis demonstrates a steady growth of patient visits to the emergency ward over the studied ten-year period (Fig. 1).

The progressive increase in patient visits to the psychiatric emergency ward, illustrated in Fig. 1, reveals a consistent upward trajectory over the entire ten-year study period, with an average annual growth rate of 4.4% (Table 1). The red dotted line representing the maximum capacity threshold, defined by the clinic, serves as a critical reference point, indicating that the number of daily patient visits exceeds this threshold more frequently, reflecting a concerning trend of recurrent overcrowding.

### Frequency of delirium and psychosis over time

**Fig. 2:**
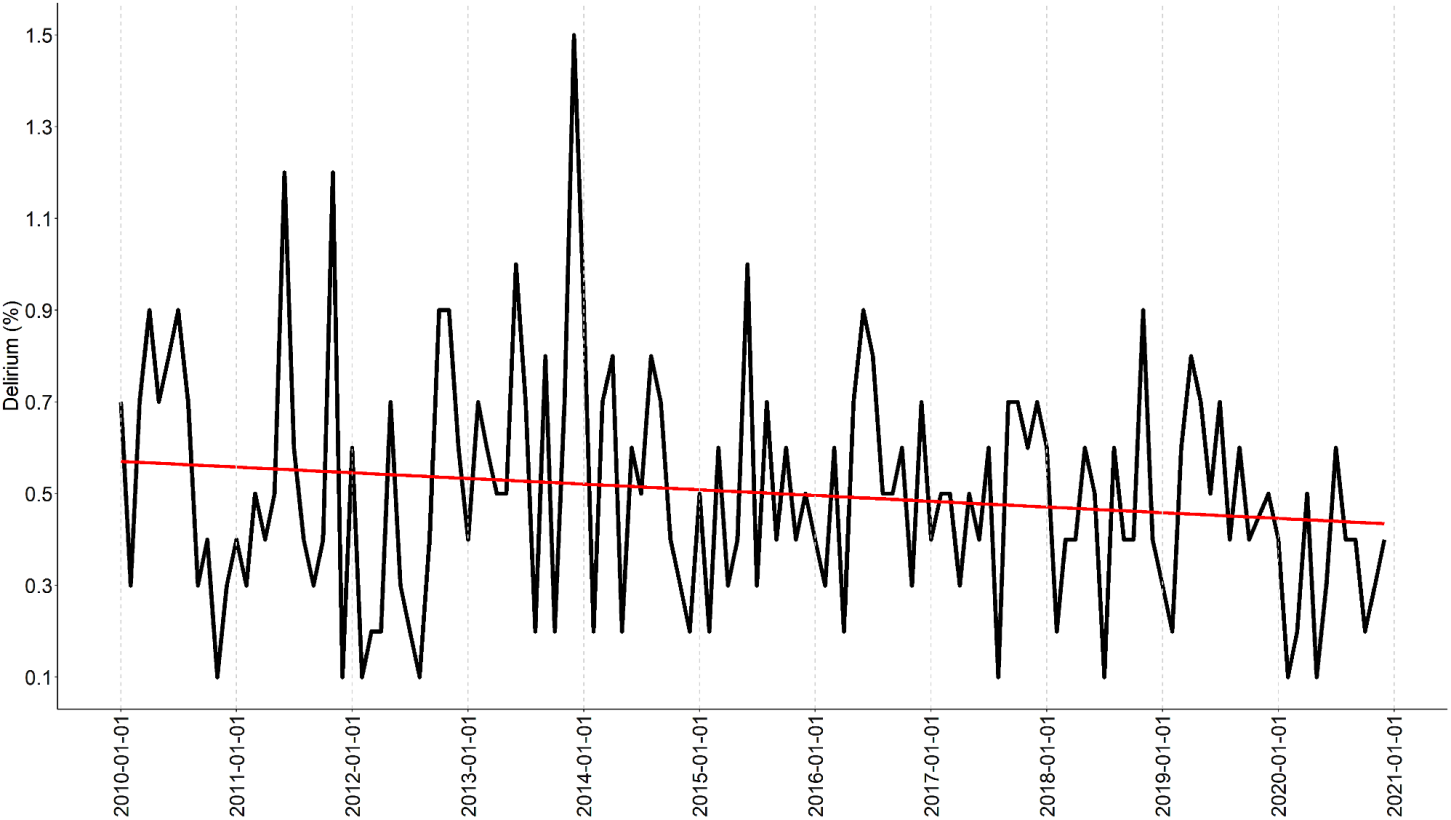
Proportion of patients seeking care who received a diagnosis of delirium tremens (F10.4) when visiting the emergency ward

**Fig. 3:**
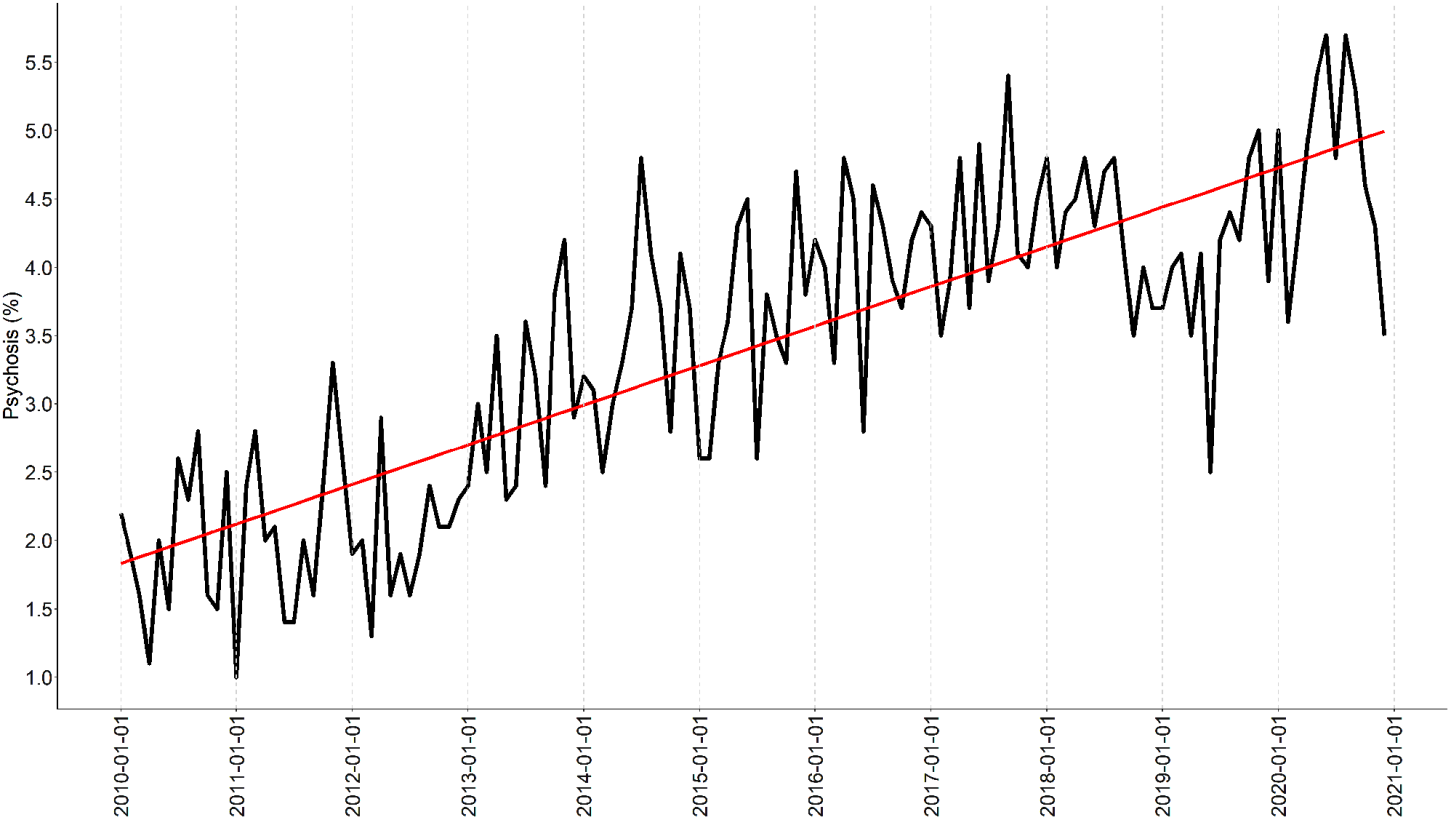
Proportion of patients seeking care who received a diagnosis of psychosis (F10.5, F11.5, F12.5, F13.5, F14.5, F15.5, F16.5, F17.5, F18.5, F19.5 and F29.9) when visiting the emergency ward

The findings demonstrate a decrease in patients being diagnosed with delirium when visiting the emergency ward (Fig. 2). This is in contrast with the percentage of patients receiving a psychosis diagnosis, which increased from around 2% in 2010 to over 5% in 2020 (Fig. 3).

The longitudinal analysis demonstrates a decrease in delirium tremens diagnoses, falling from 0,6% in 2010 to 0,5% in 2020 indicating a change in these complex conditions. In contrast, the proportion of patients diagnosed with psychosis, shown in Fig. 2, exhibited an increase from approximately 1,75% in 2010 to 4,75% in 2020, indicating a shift in the clinical characteristics of patients seeking care to the emergency ward.

The focus group was presented with the result from Figs. 1, 2 and 3; Table 2 shows the results from the focus group; key words and statements have been listed to form theme findings.

**Table 2:** Focus group on patient safety

Main findings	Theme
**Challenges for patient safety**	
1. Transport to medical facilities	Risk for delayed treatment
2. Transporting patients to other ward units requires two healthcare workers, which blocks intake of new patients	(1 + 2 + 3 + 4 + 5)
3. Moving one patient from a subsequent ward unit to another block transitions from the emergency ward	
4. Treatment for patients with delirium tremens or psychosis	Risk for increased violence
5. Insufficient staffing	(5 + 6 + 7 + 8)
6. Crowded ward unit, several patients at the same time	
7. Violence and threats from patients	
8. Continuous observation	
9. Patients with complex somatic and psychiatric needs	Risk of underestimating needs
10. Lack of information to the patients in the emergency ward increases time for enrolment in the next ward unit	(8 + 9 + 10 + 11)
11. Outgrown the premises	
**Strategies to manage pressure**	
1. First-line chief of staff works clinically, leads to increased understanding of front-end workers’ needs	Accessible manager (1 + 2 + 3 + 5)
2. Asking colleagues “what do you need?” instead of monitoring and controlling	
3. Accessible doctors	
4. It’s easier to say no for the subsequent ward unit if all their beds are occupied	Prioritising treatment (4 + 6 + 7 + 10)
5. Low hierarchy—anyone can request assistance for a patient in need	
6. Workload is not constant—this helps front-end workers recuperate	Innovative competence
7. When ward is overcrowded, doctors can be called with the specific aim of discharging someone	(8 + 9 + 10)
8. All workers have increased their competence in acute somatic healthcare	
9. Somatic assessments are made in the psychiatric ward more frequently	
10. Treatments can be given in the hallway and the corridor	

The focus group discussions, comprising five clinical experts from the ward, elucidated that one of the primary patient safety challenges involves the time-consuming process of transporting patients to medical facilities for further assessment and treatment, sometimes leading to prolonged waiting times for other patients. The requirement for two healthcare workers to accompany patients during transport, resulting in staff shortages, was identified as a recurring obstacle to efficient patient intake in the emergency ward. The need for smoother transitions between different ward units and strategies for optimizing patient flow emerged as key areas of concern, as bottlenecks and delays were identified during these transitions, which in turn strained available resources.

The combined pressures of overcrowding, risk of violence, and the management of patients with complex conditions have prompted the front-end workers to develop innovative strategies, such as increased competence in acute somatic care and empowering all staff members to request assistance when needed, enabling them to effectively address the dynamic challenges presented by patients at need.

### Staffing and beds

The ward was constructed in 2009, and between 2010 and 2014 there was room for seven patient beds. After 2014, this increased to nine patient beds. Staffing has fluctuated over the years, and the organisation has no measurement of variation between nurses, doctors and nurse-assistants’ employment. In 2015, the management agreed that 55 patient visits in one day would constitute maximum capacity, under which working conditions would be considered stretched.

## Discussion

The focus group expressed that overcrowding caused frustration and dissatisfaction among the patients, sometimes causing conflicts both with fellow patients and against the staff (Table 2). Fig. 1 indicate that such consequences could be anticipated as the management had defined a stressed condition when assessing 55 patients in one day, suggesting emergent patterns of risk [4].

### Increasing demand

The result revealed an increasing demand near the wards own definition of max capacity, and the focus groups perceived increasing workload was confirmed by an increase in the proportion of patients with an diagnose of psychosis, from 1,75% to an average of almost 5% during the studied period (Fig. 2). This introduces complex patient safety issues as patients with psychosis require a high level of care and attention. Even though workload pressure can result from various factors, such as high patient volume, limited staffing and complex patient cases (Table 2). This study confirmed the staff’s perception of an increase in patient visits over time, average annual growth rate of 4.4% (Table 1), and it could be expected that the daily average of patient visits will meet the ward’s definition of max capacity in the next couple of years (Fig. 1).

A large number of patients in a psychiatric emergency ward can lead to patients not receiving timely treatment and assessments, which can increase their risk of preventable harm [35]. Table 2 indicated a risk of delayed somatic diagnoses and follow-up care. Previous studies show that overcrowding have effects on mortality, timely treatments, and patient discharge [12], which could be countered with additional staffing, hospital bed access, and measures that should increase efficiency [36]. The increase in beds from 7 to 9 in 2014 could account for at least some of the increase in visits over time, however, the trend analysis indicates the importance of including patient visits over time when evaluating the prerequisite for patient safety.

Viewing healthcare as a complex organisation, patient safety risks should not be deconstructed to only target individual front-end workers behaviours [37]. Risk within a system emerges from the everyday, normal interactions between patients and front-end workers [3–5]. Monitoring patient visits and system pressure over time and adjusting to accommodate changing demands [4] could be a way to increase patient safety without targeting front-end workers daily routines and procedures.

The focus group raised concerns about increased workload pressure and overcrowding as staff are required to manage complex cases while still providing care to other patients. This corresponds with studies suggesting a decrease in performance and increase in adverse clinical outcomes as a consequence of overcrowding [14]. When front-end workers are under pressure, they may be more likely to make mistakes or overlook important details. The findings in this study indicated an adaptive capacity [4] by front-end workers to cope with increased pressure in which all healthcare workers needed more competence in acute somatic conditions. As the studied ward specialises in substance use treatment, this raises concerns about possible delayed or incorrect somatic diagnoses, missed medications or a lack of follow-up care. The findings showed that emerging patient safety risk could include increase in patient volume, limited staffing or increase in complex patient cases. However, as the competence of front-end workers grew, so did the workers’ ability to manage the increased workload pressure. This suggests a level of resilience aimed at preserving patient safety [38], signifying the capability to adapt and recover from mounting challenges. Even though all systems have some range of adaptive capacities [20], the study indicated delayed treatment as a repercussion of heightened workload pressure.

### Risk as an emergent property

The result from this study indicate that front-end workers increase their competence in acute somatic treatment due to changing demands. This correspond to the notion that safety is a continually adaptive property, serving to bridge sources of safety gaps under performance pressure [39].

This study uncovered an increase in patients diagnosed with psychosis (Fig. 2). Such insights has potential to design supportive interventions for front-end workers [40]. Psychiatric patient safety management should not solely rely on the ward unit’s ability to adapt to changing demands, but instead provide support to cope with stressed conditions. Patient safety can be jeopardised if conditions overwhelm the front-end workers, diminishing their ability to detect and adjust to increasing pressure [18, 21]. The result in this study indicate that the psychiatric clinic could monitor patient visits and trends in diagnosis as a signal to detected emergent patient safety risk, thereby capture gradually changing migration towards an increasing risk of accidents.

In a review of resilient performance in emergency departments, a variety of strategies was highlighted, all sharing the common theme that these departments possess the ability to make sense of disruptions and mobilize control measures to mitigate them [41]. However, resilient organizations with an ability to adapt to increased pressure could buffer a risky work environment [22]. Risk can also be concealed through gradual acceptance, [42–44], where a steady increase of patient visits, could alter our perception of risky work environment. The findings in this study could support patient safety management in psychiatry, emphasizing the importance of continuous awareness of risk as an emergent property [5, 6].

A systematic review examining models for forecasting the number of emergency department visits indicates that such models could prove valuable for large data sets in assessing future needs [45]. Utilizing knowledge about patient visits could enhance a supportive management tool that alerts patient safety management to emerging risks. Evaluations of automatic forecasting to predict the number of monthly emergency department visits suggest that these predictions offer a valuable estimate of monthly visits one year in advance [46]. Support tools incorporating forecasting models could be a valuable technique for evaluating risks associated with patient visits over time.

### Limitations

In a psychiatric emergency ward, workload pressure is a complex issue, and it is influenced by multiple factors, including the availability of resources, the number of front-end workers and the status of the patients being treated. Using a case study limits the generalisability of the specific findings as they may not be applicable to other settings. The definition of stressed conditions came from clinical workers in the studied setting, which implies that other emergency wards may have different challenges. However, the findings can be used to explore complex phenomena and should be used to provide insights into underlying processes, such as the emergence of risk. The findings can also be used to generate hypotheses for further research.

## Conclusion

This paper aimed to explore patterns of risk emergence in a psychiatric ward and provide insights into the dynamics of workload pressure. This study indicated that increased workload pressure can be predicted at a system level by analysing patient visits and diagnostics trends over time. These findings contribute to the understanding of risk as an emergent property in patient safety management. The study advocates for ongoing awareness of patient safety risks by monitoring factors identified by clinical front-end workers as potential sources of risk. Healthcare management could employ supportive tools to detect and address emerging risks, including expected workload, overcrowding, staffing issues or bed shortages. Patient safety management should include continuous monitoring of organizational risk over time.

## Data Availability

The data that support the findings of this study are available from Health Care Services Stockholm County, but restrictions apply to the availability of these data, which were used under license for the current study, and so are not publicly available. Data are however available from the author upon reasonable request and with permission of Health Care Services Stockholm County.
